# Gibberellic Acid (GA_3_) Applied to Flowering *Heracleum sosnowskyi* Decreases Seed Viability Even If Seed Development Is Not Inhibited

**DOI:** 10.3390/plants11030314

**Published:** 2022-01-25

**Authors:** Tautvydas Žalnierius, Vaidevutis Šveikauskas, Pedro J. Aphalo, Virgilija Gavelienė, Vincas Būda, Sigita Jurkonienė

**Affiliations:** 1 Nature Research Centre, Institute of Botany, Akademijos Str. 2, 08412 Vilnius, Lithuania; vaidevutis.sveikauskas@gamtc.lt (V.Š.); virgilija.gaveliene@gamtc.lt (V.G.); vincas.buda@gamtc.lt (V.B.); sigita.jurkoniene@gamtc.lt (S.J.); 2 Faculty of Biological and Environmental Science, University of Helsinki, Viikinkaari 1, 00790 Helsinki, Finland; pedro.aphalo@helsinki.fi

**Keywords:** Sosnowsky’s hogweed, invasive species, gibberellin, seedless, dry fruit

## Abstract

Sosnowsky’s hogweed (*Heracleum sosnowskyi* Manden.), an important invasive species in Eastern Europe, is a monocarpic perennial plant that propagates exclusively by seeds. Hence, interfering with seed viability could help control its spread. In the present study, we investigated the effect of exogenous GA_3_ (25, 100 and 150 mg/L) sprayed twice onto flowering *H. sosnowskyi* plants on the development of fruits (mericarps) and their ability to germinate under field conditions over the growing seasons of 2018 and 2019. Mericarps from plants sprayed with GA_3_ failed to develop normally. The width/length ratio of mericarps decreased by 23% to 25% after 150 mg/L GA_3_ application and their average weight decreased between 7% and 39% under all GA_3_ treatments. X-ray radiographs revealed that the internal structure was malformed, with many of the mericarps lacking well-developed seeds. Proportionally fewer well-developed mericarps were produced by GA_3_-treated plants than water-sprayed control plants in 2018. Seed germination assessed outdoors in seeds buried in the ground was also severely reduced (from 58% to 99% after 150 mg/L GA_3_ application). This indicates that exogenous GA_3_ sprays result in incomplete seed development and a consequent decrease in viability and germination. As the highest GA_3_ dose used resulted in significantly reduced propagation of Sosnowsky’s hogweed through seeds in the field, GA_3_ provides a promising approach to the control of the spread of this invasive weed species.

## 1. Introduction

Invasive alien plant species (IAS) are a significant environmental challenge as they are a major threat to biodiversity with consequences for human wellbeing due to phototoxicity [1,2]. Detection of IAS and their eradication are key goals of the 2020 Biodiversity Strategy of the European Union [3].

Sosnowsky’s hogweed (*Heracleum sosnowskyi* Manden.) is one of the three giant plant species in the genus *Heracleum* (Apiaceae) including *H*. *mantegazzianum* Sommier and Levier, and *H. persicum* Desf. Ex Fischer which has invaded 19 EU countries [4,5]. These species are included in the List of Invasive Alien Species of Union Concern and national IAS lists of many EU countries [6,7,8]. Currently, mechanical and chemical methods are used to control these giant *Heracleum* species. Recently, Jodaugienė et al. [9], showed that a herbicide mix of tribenuron-methyl, triclopyr and metsulfuron-methyl can eradicate *H. sosnowskyi*. However, the use of these herbicides is restricted in some areas, e.g., in the proximity of watercourses, at the edges of the forests, etc., due to environmental safety issues. Hence, less environmentally harmful but easy to apply measures to combat giant *Heracleum* species are needed.

Sosnowsky’s hogweed is a herbaceous short-lived perennial plant that can reach 3 m in height and often forms dense monostands, thus damaging native plant communities by displacing the native flora [10,11]. Being a monocarpic plant, Sosnowsky’s hogweed propagates exclusively by seeds produced once in its life. One matured plant can produce on average 15,000–20,000 seeds per season [10,12], with most of them (95.2%) germinating and decaying in the next spring [13], thus not building a long-lived seed bank in the soil. Its persistence and spread depend on the production of viable seeds [11,14,15,16,17]. Based on these features, the use of growth regulators disrupting seed development should be effective in impeding the propagation of Sosnowsky’s hogweed through seeds.

Natural and induced parthenocarpic fruit development has been observed in different plant species. In nature, parthenocarpic fruit development may have an adaptive role by reducing seed predation. For instance, *Pastinaca sativa* L., another plant from *Apiaceae* family, produces seedless fruits to decoy herbivore caterpillars and thus defend seeded fruits [18]. Artificial parthenocarpy in fleshy fruits is often induced by the application of exogenous auxins and gibberellins on flowers before anthesis to obtain seedless fruits for commercial purposes [19]. In some cases, to increase parthenocarpic fruit sets and their growth, apical bud and axillary shoots are removed [20,21]. The ability of exogenous gibberellic acid (GA_3_) to induce parthenocarpy is often accompanied by changes in fruit morphology and histology [22,23,24]. Moreover, exogenously applied GA_3_ can reduce the number of seeds in fruits, even when flowers have been fertilized/pollinated [25,26].

It has been shown that in Sosnowsky’s hogweed, a single application of exogenous GA_3_ to satellite umbels together with mechanical manipulations (terminal umbel decapitation, axillary buds removement and prevention of cross-pollination) which enhanced the GA_3_ effect, can cause deformations in fruits, slow down embryo development and decrease seed viability assessed as germination [21]. However, as a more detailed study of the disturbances in seed development induced by exogenous GA_3_ application on intact plants is still lacking, alternative mechanisms for the GA_3_-induced impairment of germination cannot be ruled out. The present study aimed to figure out double GA_3_ treatment capability to alter mericarp development and decrease seed viability without mechanical manipulations for plants.

In the present work, the morphological changes in Sosnowsky’s hogweed mericarps from satellite umbels after two exogenous applications of GA_3_ in the field were evaluated. The degree of development of seeds in mericarps including the presence of endosperm and its relation to germination percent under field conditions was assessed.

## 2. Results

### 2.1. Effect of GA_3_ on the Morphology of Mericarps

Exogenous GA_3_ significantly decreased mericarps’ width much more than their length, as reflected in the width/length ratio consistently in both years, although seeds were on average larger in size in 2019 than in 2018 (Table 1 and Table 2). In the case of seed weight, a strong decrease in weight was observed in 2018. In this year, weight of seeds from treated umbels was decreased by 36%, 34% and 39% by GA_3_ at 25, 100 and 150 mg/L, respectively, whereas in 2019, weight was decreased only by 7% by GA_3_ at 150 mg/L (Table 1 and Table 2). It is worth mentioning that the seeds’ weight was also higher in 2019 than in 2018.

### 2.2. Effect of GA_3_ on Seed Development

**Figure 1:**
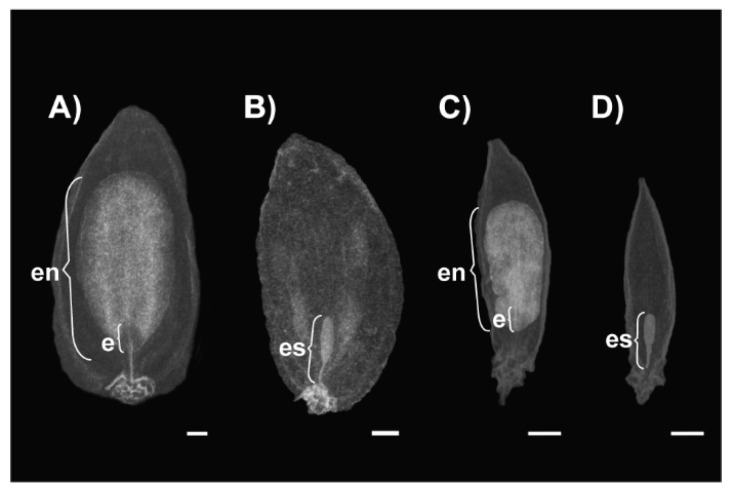
X-ray radiograph showing 100 mg/L GA_3_ effect on *H. sosnowskyi* mericarp development. (**A**) mericarp from control plant bearing fully developed seed; (**B**) seedless mericarp from control treatment sample; (**C**) deformed mericarp from 100 mg/L GA_3_ treated plant with partially developed endosperm structure; (**D**) seedless mericarp from 100 mg/L GA_3_ treated plant. Abbreviations: e—embryo; en—endosperm; es—embryo sac. Scale bar, 1 mm.

The effect of GA_3_ treatment on the internal structure of mericarps from 2018 was investigated using X-ray imaging. Radiographs revealed abnormal fruit development after GA_3_ treatment (Figure 1). The internal structure of most mericarps from GA_3_ treated umbels was altered, most notably in the endosperm and embryo, which became sac-like and almost transparent (Figure 1D). No embryo or vestige was visible in basal region of many of the mericarps. However, some mericarps developed endosperm with aberrant appearance under GA_3_ treatment (Figure 1C). Interestingly, degenerated seed structures were also observed within some mericarps from control treatment (Figure 1B).

**Figure 2:**
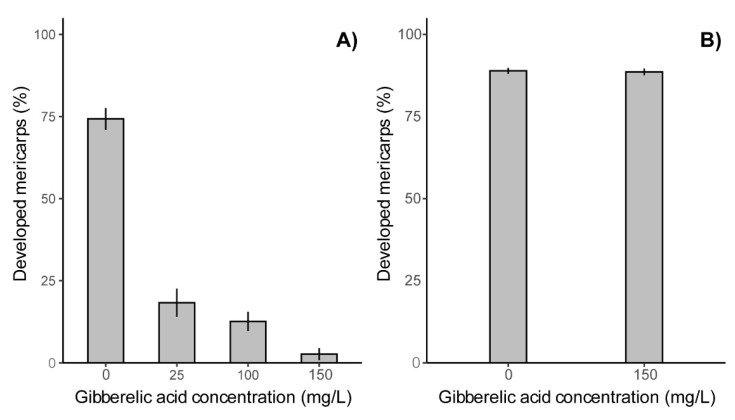
Effect of double GA_3_ treatment on the development of Sosnowsky’s hogweed mericarps over 2018 and 2019 seasons. (**A**) Data from the 2018 harvest; (**B**) data from the 2019 harvest. Data are expressed as percentage of developed mericarps per treatment. The significance of the difference between the treatments was tested using the χ^2^ test in a generalized linear model fit, (**A**) df = 3, df residuals = 456; *p* < 0.001, (**B**) df = 1, df residuals = 2129, *p* = 0.88.

Furthermore, the proportion of fruits containing developed seed structures significantly differed between satellite umbels from control and GA_3_ treated plants. In 2018 treatment with GA_3_ (25, 100 and 150 mg/L) resulted in a significant dose-dependent reduction in the percentage of fruits with normally developed endosperm (Figure 2A). These decreases were substantial and when expressed relative to controls were 75%, 83% and 97% for 25, 100 and 150 mg/L GA_3_ treatments, respectively. However, in 2019 a similar decrease was not observed in the same experimental field (Figure 2B). Nevertheless, observations from 2018 demonstrated that exogenous GA_3_ can strongly suppress *H. sosnowskyi* seed development in satellite umbels in some years. This effect of exogenous GA_3_ on mericarp development suggests that this treatment will also decrease the percentage of viable seeds produced, and their apparent germination success.

### 2.3. Effect of GA_3_ on Seed Germination in the Field

**Figure 3:**
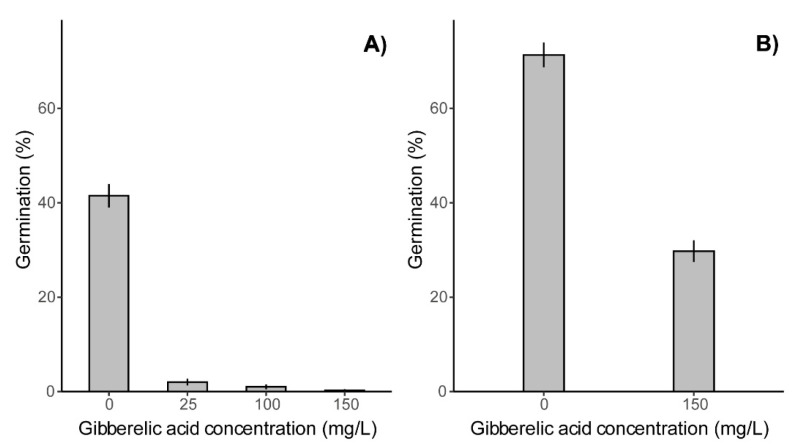
The effect of double GA_3_ treatment on Sosnowsky’s hogweed seed germination in the field. (**A**) germination of seeds from the 2018 harvest; (**B**) germination of seeds from the 2019 harvest. Data are expressed as percentage of germinated mericarps per treatment. The significance of the difference between the treatments was tested using the χ^2^ test in a generalized linear model fit, (**A**) df = 3, df residuals = 1596, *p* < 0.001, (**B**) df = 1, df residuals = 698, *p* < 0.001.

Seed germination assessed outdoors in the ground revealed that GA_3_ treatment strongly decreased seed germination in both years, although more efficiently in 2018 than in 2019 (Figure 3). Compared with the control germination decreased in 2018 by 95%, 98% and 99% with 25, 100 and 150 mg/L GA_3_ treatments, respectively (Figure 3A). In 2019, germination decreased by 58% in response to 150 mg/L GA_3_ treatment to umbels (Figure 3B). The reduction in germination was stronger in both years than what could be expected, solely based on the morphological assessment of damage.

## 3. Discussion

Impaired seed development induced by biologically active compounds, such as natural plant growth regulators, and could be used as a basis for environmentally safe weed control. In species whose persistence and spread depend on short-lived seeds, interfering with the development of fruits and seeds to reduce the number of viable seeds should have a strong effect on further weed spread. Such an approach could be useful with the invasive monocarpic weed species *H. sosnowskyi*, but a protocol remains to be developed and its efficiency demonstrated. Results from our earlier studies with this species suggest that the disturbance of mericarp development induced by exogenous GA_3_-application can contribute to decreased germination [21]. Meanwhile, in the current study, the double application of GA_3_ shows a dose response and consistent efficacy in reducing seed viability in different years. Moreover, from a practical point of view, such an application of GA_3_ allows for replacing the mechanical manipulations by spraying flowers at a phytohormone-sensitive stage of development [20,21].

In many species, normal fruit development is dependent on the development of seeds inside them. For example, in apple cultivars, the percentage of fruits with deformations decreased with the increase in the number of seeds per fruit [27]. In many cases, the role of seeds in fruit development is related to phytohormone synthesis in seeds [23,28,29,30]. In addition to fruit size, the final shape of fruits is under hormonal control and is dependent on the development of seeds [31]. For example, exogenous GA_3_-induced seedlessness in seeded grape cultivars had a concurrent effect on fruit morphology [32].

Thus, we assessed if exogenous GA_3_ can induce morphological alterations to the mericarps of Sosnowsky’s hogweed. We observed that GA_3_ treatment resulted in mericarps with similar length but decreased width. A similar decreased width/length ratio after exogenous GA_3_ application is common in fleshy fruits (expressed as length/width ratio in [25,33,34,35]. A possible explanation for such a change in fruit shape is a decrease in ovary width due to the lack of seeds [34,36]. Alternatively, the change in shape could be due to differences in sensitivity to exogenous gibberellins of various tissues in flowers and fruits [33,35]. However, some species deviate from this usual pattern. One of them is *Pastinaca sativa*, another member of *Apiaceae* which can naturally produce seedless fruits that are similar in appearance to fruits containing well-developed seeds [18]. Our observations show that this is also true for naturally occurring seedless fruits in untreated Sosnowsky’s hogweed plants (Figure 1B). This indicates that at least in some *Apiaceae* species fruit development may continue normally, even in the absence of developing seeds. From this, it follows that exogenous GA_3_ application could induce morphological changes in the mericarps of Sosnowsky’s hogweed independently of seed development, which was confirmed by the radiographic images (Figure 1C,D). This result suggests that differences in sensitivity to exogenously applied GA_3_ of the various tissues of the future fruit played an important role. It should be noted, however, that in an earlier experiment using the same species the impact of exogenous GA_3_ on fruit size was not observed [21]. Discrepancies with the lack of effect on mericarp size reported by Koryznienė et al. [21] and our new findings, may have been the result of the GA_3_ treatment having been applied once in the earlier study and twice in the current study. Interestingly, the decrease in mericarps width/length ratio after 150 mg/L GA_3_ treatment was very similar in 2018 and 2019 years, despite the fact that, on average, fruits were larger in 2019. This clearly demonstrates that tissues of the future fruit were similarly susceptible to GA_3_ in both years.

The altered shape of fleshy fruits after GA_3_ application is frequently accompanied by a decrease in fruit weight [25,33,34]. Cheng et al. [32] observed decreased fruit weight in seeded grape cultivars after exogenous GA_3_ application but increased weight in seedless ones. In our study, the average weight of Sosnowsky’s hogweed ripe mericarps decreased in response to GA_3_, more in 2018 than in 2019 (Table 1 and Table 2). The fruit weight decrease can be attributed, at least in part, to aborted seeds (particularly missing endosperm) in 2018, but in 2019 the smaller decrease in mericarp size and weight may have depended on a different mechanism. Differences between years can be attributed to differences in environmental conditions during fruit development and growth and could have also affected the previous study. The clear differences in the size and weight of mericarps produced by control plants in 2018 and 2019 suggest that weather conditions may have played an important role.

As discussed above, exogenous GA_3_ treatment decreased fruit size, whereas without treatment seedless fruits naturally produced retain their normal size and morphology. As in earlier studies, exogenous GA_3_ applied to induce parthenocarpy has concurrently induced changes in fruit morphology and histology in multiple plant species [22,23,24], we expected that GA_3_-induced changes in the external morphology of mericarps would correlate with internal changes (missing or vestigial endosperm and embryo). The absence of seeds in fleshy fruits after the treatment of young flowers with hormones has been frequently reported [19,28,36,37,38]. Moreover, the molecular mechanisms of hormone regulation on fruit set appear to be similar between fleshy and dry fruits [24]. X-ray images of *H. sosnowskyi* mericarps revealed an effect of GA_3_ on their internal structure (Figure 1C,D): exogenous GA_3_ application on flowers, including exposed ovaries, before anthesis, prevented normal development of the endosperm and embryo, thus leading to seedless mericarps in 2018 (Figure 1D). The ability of exogenous GA_3_ to disturb Sosnowsky’s hogweed seed development seems not to depend on pollination [21].

Had the main effect of GA_3_ been blocking the formation of mericarps, then viability of a few remaining fruits would have been of little relevance. However, given that mericarps were still produced in abundance, even if smaller in size on average, makes their ability to germinate under field conditions is a decisive question. When, as in 2018, the disturbance of seed development by GA_3_ is strong, it can be assumed that decreased germination is mainly due to a high proportion of “empty” seedless mericarps being produced. An approximate estimate of the percentage of non-viable but well-formed seeds can be obtained from these values: 44%, 89%, 92% and 88%, with 0, 25, 100 and 150 mg/L GA_3_, respectively. In 2019, GA_3_ did not prevent seed development as assessed, but it did decrease germination substantially. Seeds were larger overall; even seeds from GA_3_ treated plants were noticeably larger than those from previous year’s controls. The approximate percentage of non-viable but well-developed seeds was 20% in controls but 66% in GA_3_ treated ones. These derived values suggest the idea that besides the impact on “empty” seed development, GA_3_ treatment of flowers may also induce processes related to the germination of apparently well-formed seeds.

**Figure A1:**
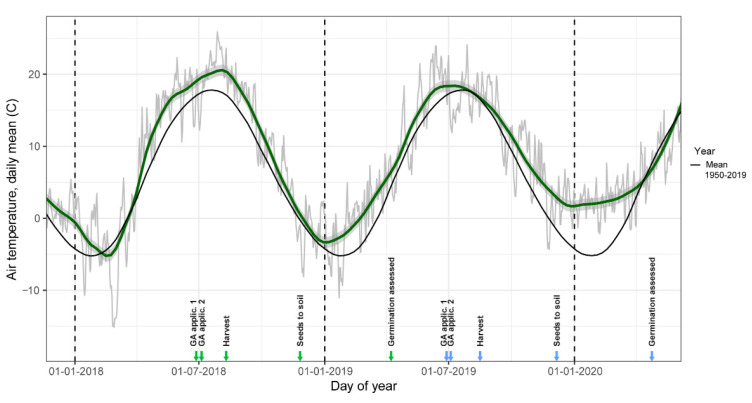
Mean temperature over the period 2018–2019 in Vilnius meteorological station, Lithuania. Data used: E-OBS dataset from the EU-FP6 project UERRA (https://www.uerra.eu) (accessed on 6 December 2021) and the Copernicus Climate Change Service, and the data providers in the ECA&D project (https://www.ecad.eu) (accessed on 6 December 2021) https://surfobs.climate.copernicus.eu/dataaccess/access_eobs.php, 21.0e edition, 0.1 deg. Regular grid data were used for figures. Accessed on 6 December 2021.

**Figure A2:**
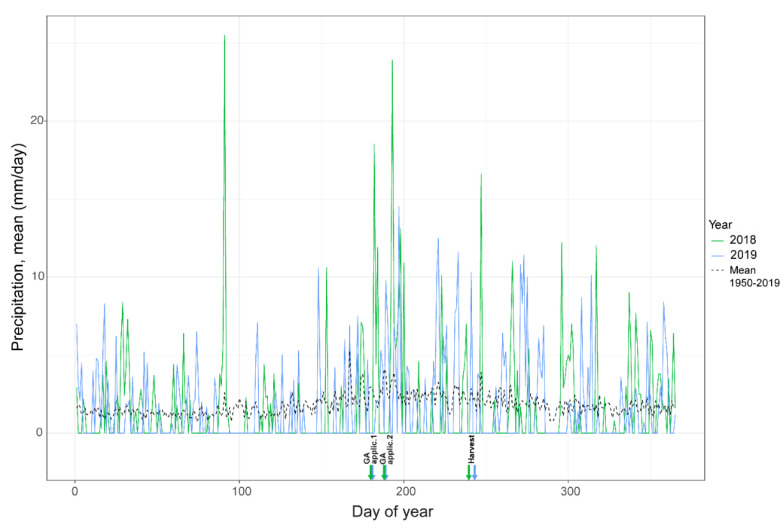
Mean precipitation over the period 2018–2019 in Vilnius meteorological station, Lithuania. Arrows indicate day of GA_3_ applications and harvest in 2018–2019. Data used: E-OBS dataset from the EU-FP6 project UERRA (https://www.uerra.eu) (accessed on 6 December 2021) and the Copernicus Climate Change Service, and the data providers in the ECA&D project (https://www.ecad.eu) (accessed on 6 December 2021), https://surfobs.climate.copernicus.eu/dataaccess/access_eobs.php (accessed on 6 December 2021), 21.0e edition, 0.1 deg. Regular grid data were used for figures.

Differences in germination, size and morphology of mericarps between the experiments conducted in 2018 and 2019 were large. The low germination percentage in the absence of treatment observed in 2018 was lower than previously reported for the same species [11,12,15,21,39], whereas that observed in 2019 was more similar to them. Variation among individual plants at the same site has also been reported earlier [40]. As not only germination but also the proportion of well-developed seeds was low in 2018, it is most plausible that the differences between 2018 and 2019, mericarp germination was determined by their development, rather than by germination conditions in the subsequent Spring. Meteorological records show some differences between 2018–2019 seasons. The springtime of 2018 was warmer and dryer than in 2019. Moreover, the weather in June–July in the period of fruit development in 2018 was warmer and more wet compared with 2019 (Figure A1 and Figure A2). These factors may be important for fruit quality because natural parthenocarpy can be induced by environmental stressors such as heat, herbivory, etc. [14,15,37,38]. In 2019, the percentage of seedless mericarps in controls was 57% lower than in 2018. Thus, these findings may suggest an idea for setting an effective GA_3_ concentration to control seed viability over different climate conditions.

## 4. Materials and Methods

### 4.1. Plant Material and GA_3_ Treatments

**Figure 4:**
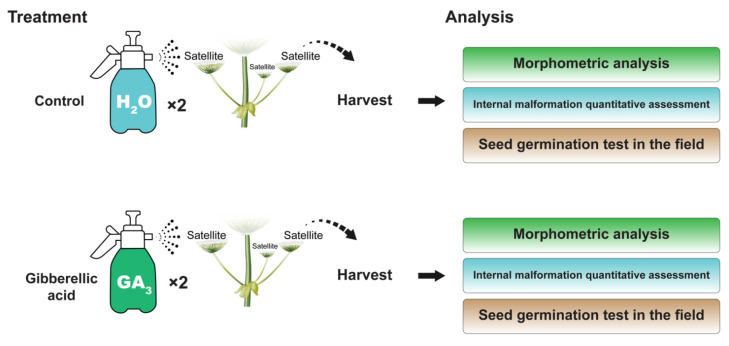
The experimental set-up.

Sosnowsky’s hogweed (*Heracleum sosnowskyi* Manden.) plants located at anthropogenic herbaceous stand close to a forest edge in Vilnius, Lithuania (Latitude = 54°44′23.1″ N, Longitude = 25°15′31.9″ E) were selected and treatments assigned at random to them. To induce seedlessness, satellite umbels producing seeds with potentially higher levels of spreading were chosen. In each plant, three randomly selected satellite umbels (umbels surrounding the primary umbel) were treated with gibberellic acid (GA_3_) (SERVA, Heidelberg, Germany) dissolved in distilled water at three concentrations (25; 100 and 150 mg/L). Treatment with gibberellic acid was applied one day before anthesis in late June in 2018 and 2019, spraying 18.5 mL of GA_3_ solution per umbel (i.e., 55.5 mL per plant) from 15–20 cm distance with backpack sprayer (KB-16E-6, KOBOLD, Xiamen, China). As flowers open at different times within an umbel, the treatment was repeated after 8 and 6 days in 2018 and 2019, respectively, to enhance its efficiency. Control plants were sprayed with the same volume of distilled water (Figure 4). Treatments were carried out on the first part of a day. On the days of treatment, rain was not observed. Mature fruits were harvested in late August both in 2018 and 2019 from 10 and 8 plants, respectively. Fruits from the sprayed umbels were pooled together by the plant. Two to four plants were used per treatment.

### 4.2. Morphometric Analysis

Ten percent of completely dry harvested mericarps from each biological replicate (100 to 600 mericarps per plant) (from one plant in GA_3_ 100 mg/L treatment 40 mericarps were observed) were randomly selected for the assessment of their morphometric parameters: length, width and weight. For the evaluation of length and width of each mericarp ruler (1 mm) was used. The individual weight of mericarps was measured with an analytical balance (WAA 160/C/1, RADWAG, Radom, Poland).

### 4.3. Internal Malformation Assessment

For qualitative analysis, 30 randomly selected mericarps were X-rayed (Faxitron MX-20, Faxitron Bioptics LLC, Tucson, AZ, USA) with CEA orthochromatic mammography film (Agfa Healthcare NV, Ghent, Belgium). Mericarps with less than 10% of their volume occupied by the endosperm were considered as seedless and otherwise as containing a seed. The degree of the development (seedless or with seed) was determined in the same 100 to 600 mericarps per replicate (from one plant in GA_3_ 100 mg/L treatment 40 mericarps were observed) used for morphometric analysis by observation under a binocular microscope (Olympus, Tokyo, Japan) with transillumination.

### 4.4. Seed Germination Test in the Field

Germination was tested in the Didieji Gulbinai experimental garden, Vilnius district, Lithuania (Latitude = 54°46′38.67″ N, Longitude = 25°17′29.81″ E). One hundred randomly selected seeds from each of 3–4 biological replicates per treatment were spread into polyethylene bags filled with fertilized peat (pH 5.5–6.5, SULIFLOR SF2, Radviliškis, Lithuania). All the bags were pierced for drainage and buried in the soil at a depth of ~5 cm in late November 2018 and in early December 2019. Bags were excavated in April 2019 and in late April 2020, respectively, and germinated seeds were counted. Mericarps with protruding radicles were considered to be germinated.

### 4.5. Statistical Analysis

Data about the effect of different GA_3_ treatments on mericarp internal structure development and seed germination were analysed by fitting generalized linear models under a binomial distribution with a logit link function. We used function glm from R 4.1.1 [41] and tested for possible deviations from assumptions with R package DHARMa [42]. With data from 2018, the first step in the analysis was to test if GA_3_ application had a significant effect by fitting a model with treatments as discrete levels of a factor. In addition to the overall ANOVA, a contrast between control and all the GA_3_ treatments pooled together was applied. As a second step, to test if increasing concentrations of GA_3_ significantly increased the response, a model was fit considering the GA_3_ concentration as a continuous variable while excluding the control. The errors reported in figures are from the model predictions. As in 2019, only two treatments, control and GA_3_ application, were applied, and only a *t*-test for the effect of GA_3_ was carried out.

Other data obtained from the 2018 experiment are for continuous variables describing mericarp morphology. For these data, the effect of the treatments was tested with one-way analysis of variance (ANOVA) in SPSS (26.0, IBM). For comparison of means, Tukey’s HSD test for significant differences at significance level *p* < 0.05 was used. Morphometric data from 2019 were analysed by comparing means using a *t*-test in SPSS. Mean values were calculated within samples for each treatment.

## 5. Conclusions

In conclusion, it was shown that double GA_3_ treatment during the flowering of invasive *H. sosnowskyi* ensured the dose response and consistency in the efficiency in decreased seed germination. Moreover, two causes for decreased viability of mericarps induced by GA_3_ applied to flowers were identified: (1) an increase in the proportion of seedless mericarps and (2) a decrease in the ability to germinate under field conditions of well-formed mericarps. The study extended over two years, and in each of these years, different mechanisms predominated. The observed reduction in seed viability after GA_3_ application will interfere with the yearly replenishment of the soil seedbank and also make further weed spread less likely. The same approach is promising in the control of closely related invasive weed species such as monocarpic *H. mantegazzianum.* Further studies including the scaling up of application methods and investigations on the mechanism of exogenous GA_3_ impact on well-formed mericarp germination in different climate conditions are needed before the approach can be applied to weed control at a commercial scale.

## Figures and Tables

**Table 1:** Effect of double GA_3_ treatment on Sosnowsky’s hogweed mericarps morphometric parameters in 2018 year’s season.

GA_3_ Concentration (mg/L)	Length (mm)	Width (mm)	Weight (mg)	Width/Length Ratio
0	6.75 ± 0.007 ^A^	4.43 ± 0.004 ^A^	6.22 ± 0.17 ^A^	0.66 ± 0.005 ^A^
25	5.76 ± 0.07 ^B^	3.38 ± 0.006 ^B^	3.96 ± 0.14 ^B^	0.59 ± 0.01 ^B^
100	6.31 ± 0.08 ^B^	3.84 ± 0.006 ^C^	4.11 ± 0.18 ^B^	0.61 ± 0.006 ^B^
150	6.79 ± 0.08 ^A^	3.39 ± 0.005 ^B^	3.78 ± 0.14 ^B^	0.51 ± 0.006 ^C^

Values are means ± SE (*n* = 2–3 biological replicates). Different letters indicate statistically significant differences at significance level *p* ˂ 0.05 (Tukey’s HSD test).

**Table 2:** Effect of double GA_3_ treatment on Sosnowsky’s hogweed mericarps morphometric parameters in 2019 year’s season.

GA_3_ Concentration (mg/L)	Length (mm)	Width (mm)	Weight (mg)	Width/Length Ratio
0	8.99 ± 0.01 ^A^	6.12 ± 0.003 ^A^	8.98 ± 0.13 ^A^	0.68 ± 0.002 ^A^
150	9.63 ± 0.005 ^B^	4.86 ± 0.003 ^B^	8.33 ± 0.13 ^B^	0.51 ± 0.002 ^B^

Values are means ± SE (*n* = 4 biological replicates). Different letters indicate statistically significant differences at significance level *p* ≤ 0.01 (*t*-test).

## Data Availability

The data supporting reported results can be found in scientific reports of the Laboratory of Plant Physiology of Institute of Botany of Nature Research Centre, where archived datasets generated during the study are included.
